# Response to the Letter to the Editor: Unlocking Better Keloid Treatment: Corticosteroid, 5‐Fluorouracil, and Hyaluronidase vs. Corticosteroid Alone: A Randomized Comparative Study

**DOI:** 10.1002/hsr2.71891

**Published:** 2026-02-22

**Authors:** Ala Ehsani, Fatemeh Lotfi, Alireza Firooz, Amirhoushang Ehsani, Zahra Razavi, Mahshid Sadat Ansari, Pedram Nourmohammadpour, Fatemeh Sima, Mina Koohian Mohammadabadi, Amirhossein Rahimnia

**Affiliations:** ^1^ Student Research Committee, Faculty of Medicine Shahid Beheshti University of Medical Sciences Tehran Iran; ^2^ School of Medicine Iran University of Medical Sciences Tehran Iran; ^3^ Center for Research & Training in Skin Diseases & Leprosy Tehran University of Medical Sciences Tehran Iran; ^4^ Department of Dermatology Razi Hospital, Tehran University of Medical Sciences Tehran Iran; ^5^ Autoimmune Bullous Diseases Research Center Razi Hospital, Tehran University of Medical Sciences Tehran Iran; ^6^ Students’ Scientific Research Center (SSRC) Tehran University of Medical Sciences Tehran Iran

We appreciate the attention to our article and the thoughtful comments provided. This study was intended as a first step toward exploring a novel approach to keloid treatment rather than relying solely on conventional conservative options.

Regarding the small sample size, our aim was to assess feasibility and preliminary efficacy in a pilot setting. At the time, there was limited scientific background supporting this combination, and based on prior experience, we anticipated challenges in convincing patients to try the novel procedure. For this reason, we preferred to conduct a smaller study rather than abandon the idea, viewing it as a starting point for future work. The limited sample size was clearly acknowledged in our discussion. We agree that reporting effect sizes would have strengthened interpretation, and this is an important consideration for subsequent studies.

When designing the trial, the literature on hyaluronidase in keloid treatment was minimal. To our knowledge, the only relevant research was by Goyal et al. [1], which evaluated the triple combination of steroid, 5‐FU, and hyaluronidase. We adopted this as the reference for our comparison. We acknowledge that this design does not allow isolation of hyaluronidase's independent effect and explicitly noted this in our discussion.

As for blinding, participants became aware of their allocation because of the presence or absence of lidocaine in the injections. Before enrollment, they were informed of the components in each group. Since lidocaine substantially reduces procedural pain, its absence or presence inevitably revealed the group. We prioritized minimizing patient discomfort over complete blinding. To reduce bias in objective measures, a blinded dermatologist documented outcomes. Nevertheless, we recognize that subjective measures such as pain and pruritus may be influenced by perception, and some degree of bias is inevitable. In future studies, standardizing the amount or route of anesthetic administration may help minimize this effect.

The short follow‐up period was another deliberate choice. In interventional scar studies, long‐term follow‐up is crucial; however, with a small cohort, the risk of missing data from patient dropout was considerable. We therefore opted for a shorter follow‐up to ensure complete data collection, which we achieved.

We recognize the limitations noted in the letter—including small sample size, short follow‐up, partial blinding, and potential confounding from multiple agents in the intervention group—and we reflected these in our discussion. Our aim was not to draw definitive conclusions, but to highlight the possible promising role of hyaluronidase in keloid management. We hope this pilot study will encourage larger, more rigorous trials to validate and expand on our findings, and we appreciate the shared commitment to advancing research in this field.

## Author Contributions

**Ala Ehsani:** data curation, investigation, formal analysis. **Fatemeh Lotfi:** investigation, formal analysis, data curation, writing – original draft, writing – review and editing. **Alireza Firooz:** conceptualization, supervision, project administration, writing – review and editing. **Amirhoushang Ehsani:** conceptualization, writing – review and editing, project administration, supervision. **Zahra Razavi:** methodology, formal analysis, supervision, validation, writing – original draft. **Mahshid Sadat Ansari:** methodology, validation, formal analysis, supervision, writing – review and editing. **Pedram Nourmohammadpour:** conceptualization, supervision, writing – review and editing. **Fatemeh Sima:** data curation, formal analysis, methodology. **Mina Koohian Mohammadabadi:** data curation, methodology, writing – original draft. **Amirhossein Rahimnia:** conceptualization, investigation, methodology, project administration, writing – original draft, writing – review and editing. All authors have read and approved the final version of the manuscript.

## Funding

The authors received no specific funding for this work.

## Conflicts of Interest

The authors declare no conflicts of interest.

## Transparency Statement

The lead author Amirhossein Rahimnia affirms that this manuscript is an honest, accurate, and transparent account of the study being reported; that no important aspects of the study have been omitted; and that any discrepancies from the study as planned (and, if relevant, registered) have been explained.

## Data Availability

The data that support the findings of this study are available from the corresponding author upon reasonable request. The corresponding author had full access to all of the data in the original study and takes complete responsibility for the integrity of the data and the accuracy of the data analysis.
